# Supplementary figures and images for: Aggregation-prone alpha-synuclein proteoforms and dysregulated molecular signatures in the vermiform appendix of synucleinopathy patients
Source: bioRxiv. 2025 Oct 7:2025.10.07.680938. Preprint. [Version 1] doi: 10.1101/2025.10.07.680938 (PMC12632551; doi:10.1101/2025.10.07.680938)

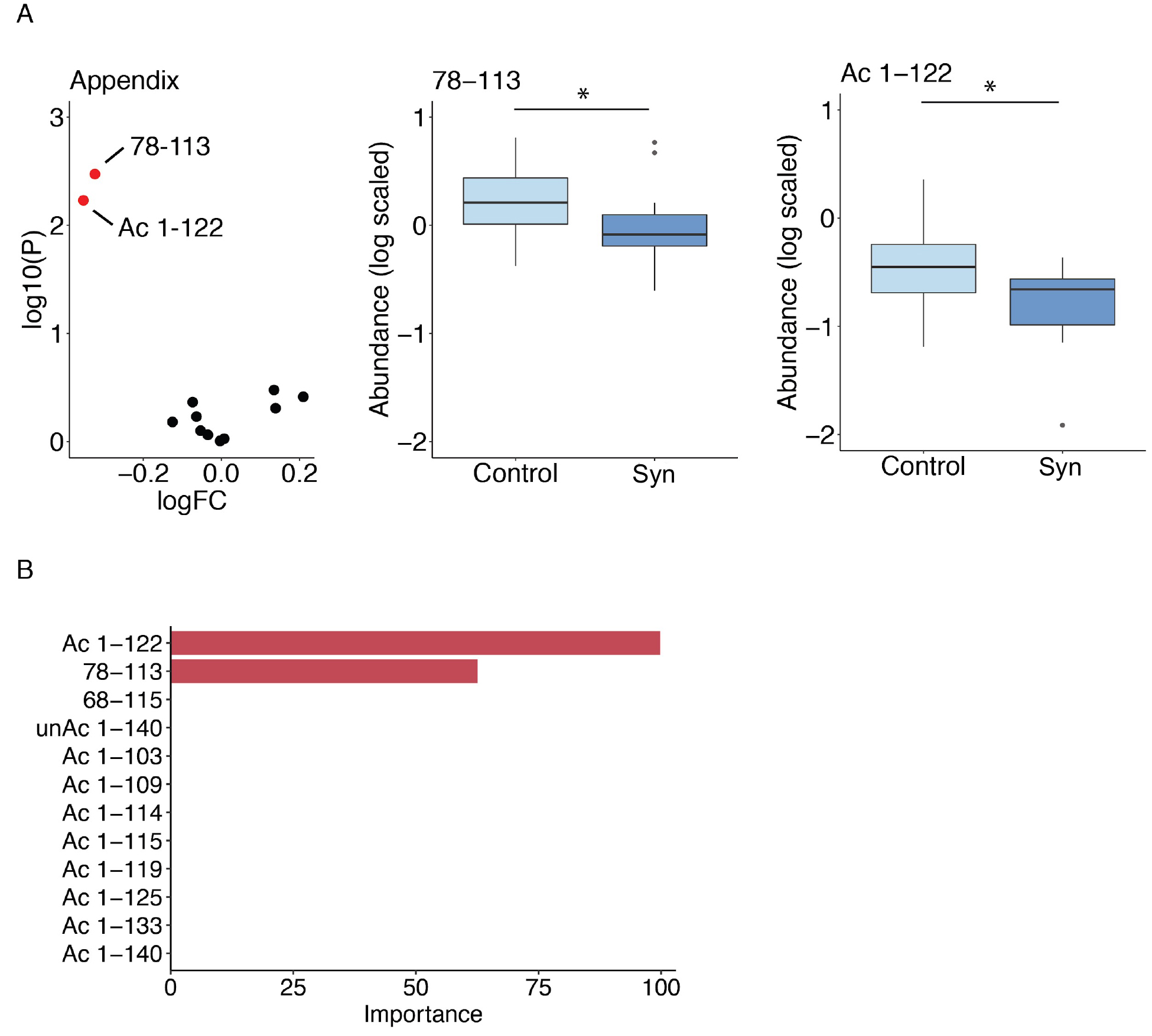

Supplement: Supplement 12 [file media-12.jpg]

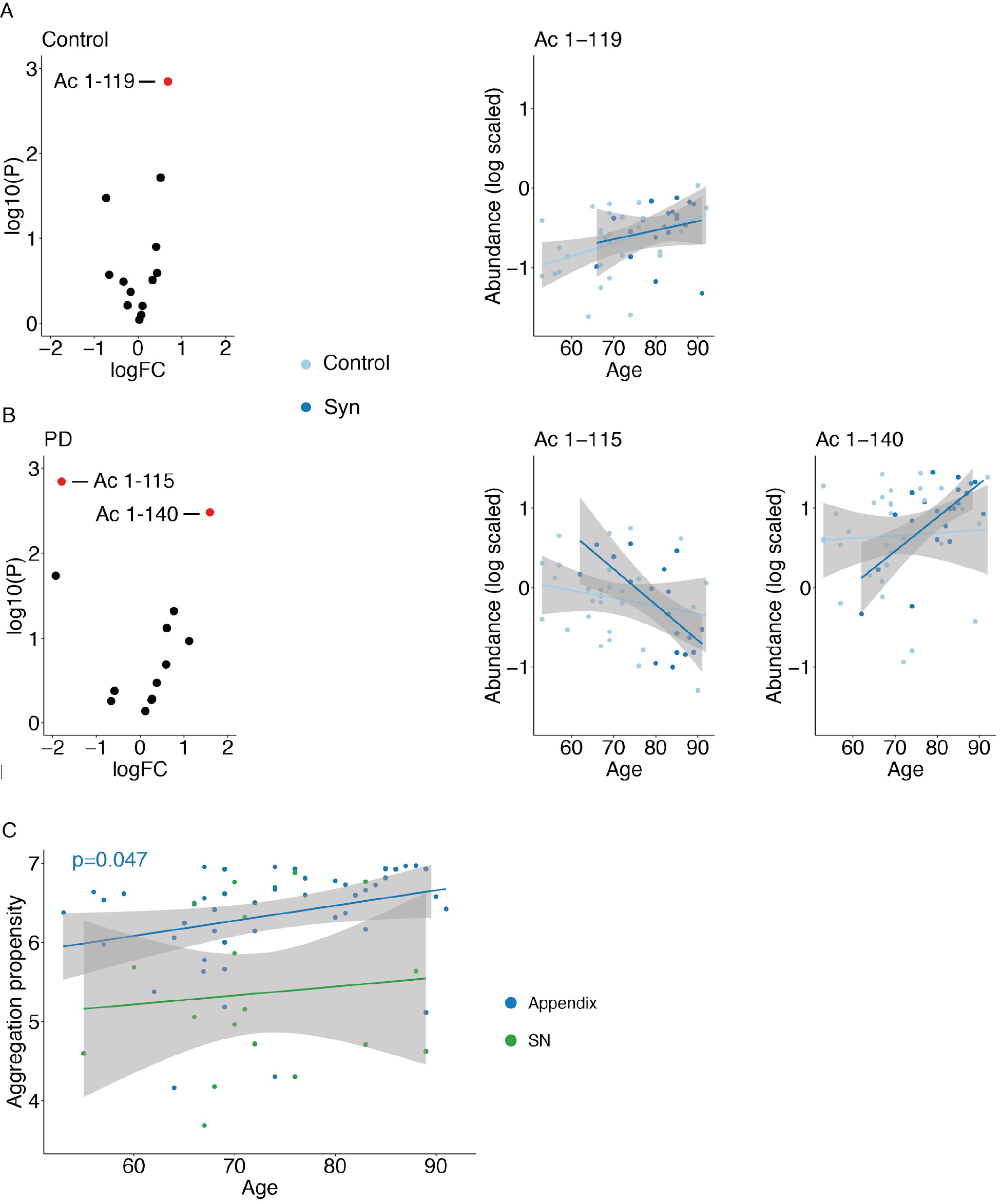

Supplement: Supplement 13 [file media-13.jpg]

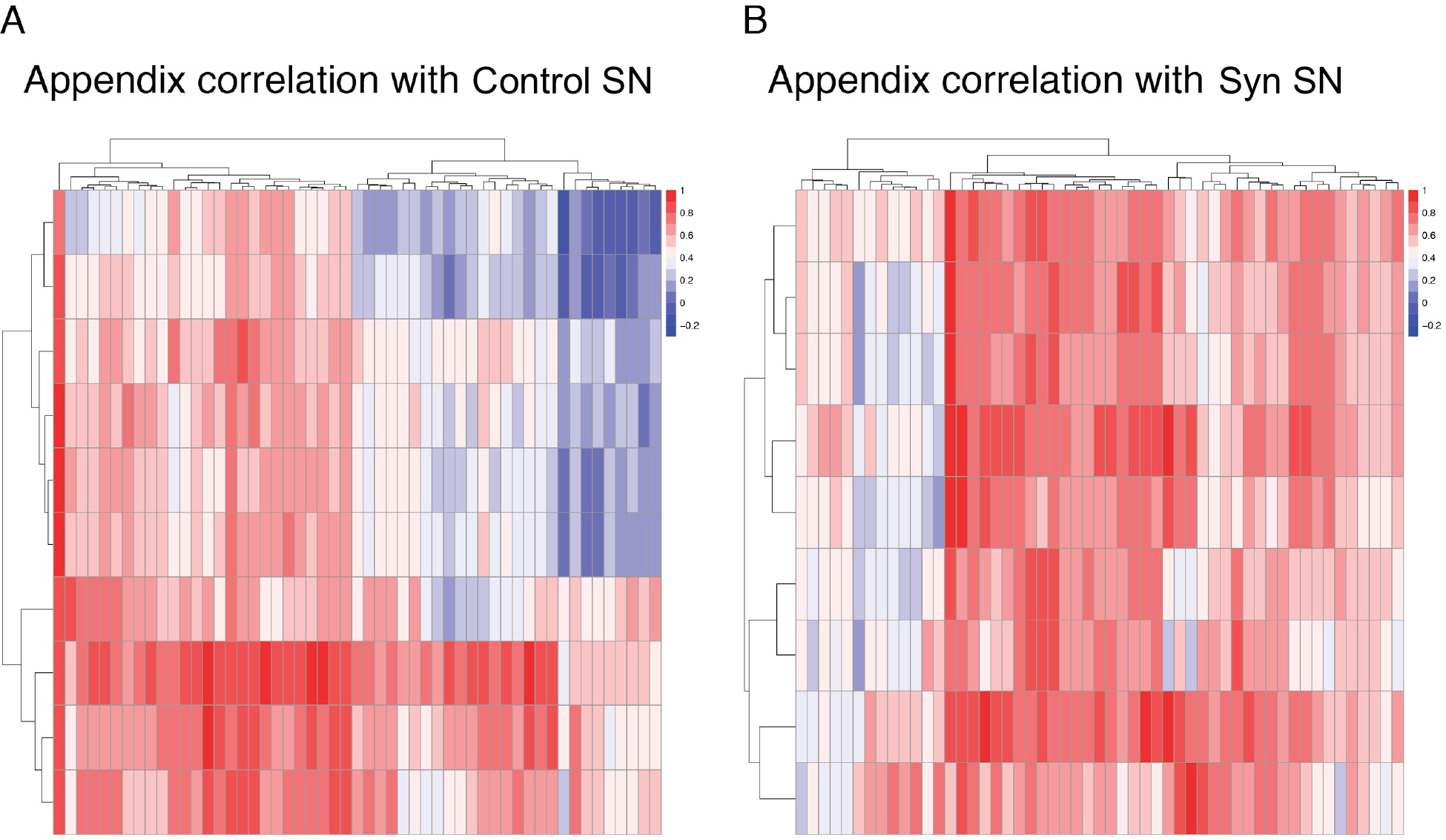

Supplement: Supplement 14 [file media-14.jpg]

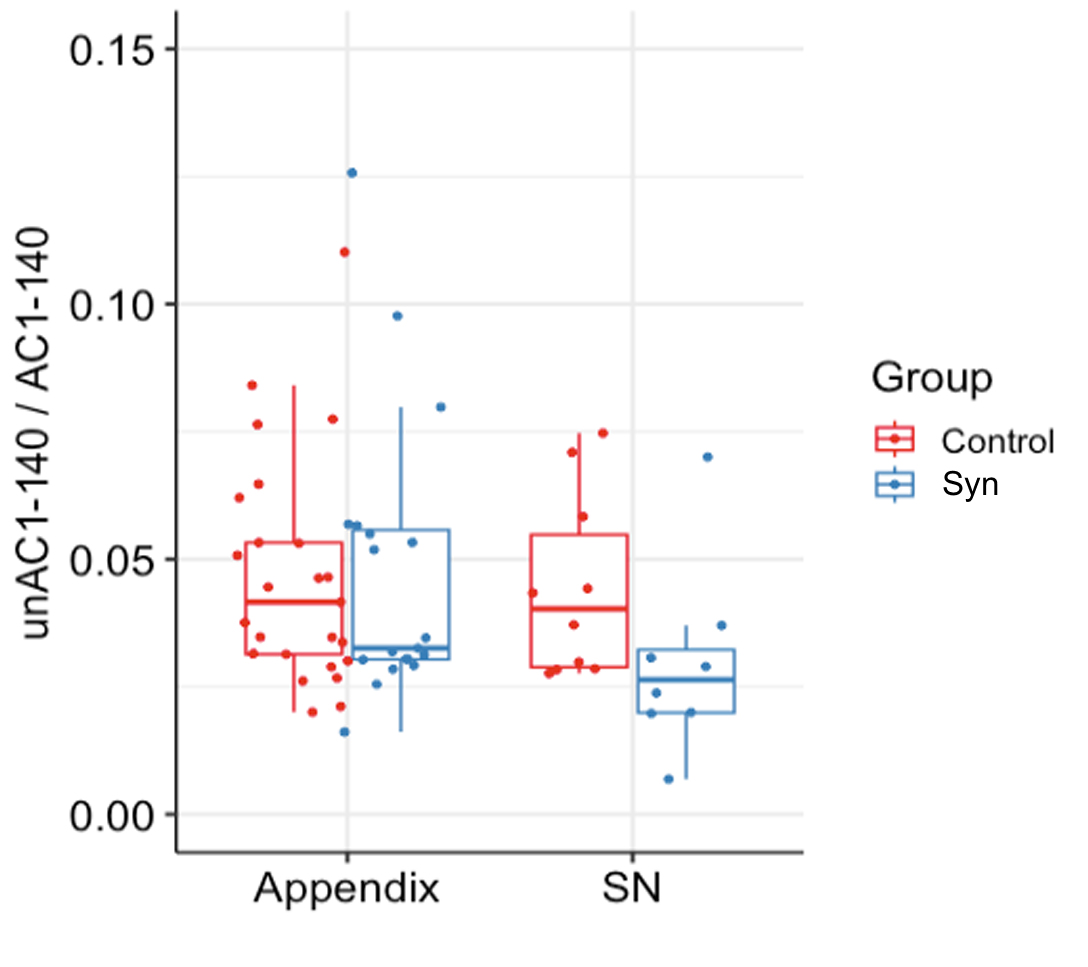

Supplement: Supplement 15 [file media-15.jpg]
